# The Global Burden of Diseases attributed to high low-density lipoprotein cholesterol from 1990 to 2019

**DOI:** 10.3389/fpubh.2022.891929

**Published:** 2022-08-16

**Authors:** Jia Zheng, Jing Wang, Yan Zhang, Jiangliu Xia, Huilan Guo, Haiying Hu, Pengfei Shan, Tianlang Li

**Affiliations:** ^1^Department of Medical Geriatrics, The Second Affiliated Hospital of Zhejiang University School of Medicine, Hangzhou, China; ^2^Department of Endocrinology and Metabolism, The Second Affiliated Hospital of Zhejiang University School of Medicine, Hangzhou, China; ^3^Department of Medical Oncology, The Second Affiliated Hospital of Zhejiang University School of Medicine, Hangzhou, China; ^4^Department of Clinical Nutrition, The Second Affiliated Hospital of Zhejiang University School of Medicine, Hangzhou, China

**Keywords:** disability-adjusted life years, Global Burden of Disease, cholesterol, low-density lipoproteins, cardiovascular disease

## Abstract

**Background:**

To demonstrate the real-word situation of burdens that are attributed to the risk factor of high low-density lipoprotein cholesterol (LDL-C) at the global, regional, national levels, among different age groups and between genders.

**Methods:**

We analyzed data from the Global Burden of Disease study 2019 related to global deaths, disability-adjusted life years (DALYs), summary exposure value (SEV), average annual percentage change (AAPC), and observed to expected ratios (O/E ratios) attributable to high LDL-C from 1990 to 2019.

**Results:**

Globally, in 2019, the total numbers of deaths and DALYs attributed to high LDL cholesterol were 1.47 and 1.41 times higher than that in 1990. The age-standardized deaths and DALYs rate was 1.45 and 1.70 times in males compared to females, while the age-standardized SEVs rate was 1.10 times in females compared to males. The deaths, DALYs, and SEV rates increased with age. In 2019, the highest age-standardized rates of both deaths and DALYs occurred in Eastern Europe while the lowest occurred in high-income Asia Pacific. High-income North America experienced a dramatic reduction of risk related to high LDL-C. Correlation analysis identified that the age-standardized SEV rate was positively correlated with Socio-demographic Index (SDI; *r* = 0.7753, *P* < 0.001). The average annual percentage change (AAPC) of age-standardized SEV rate decreased in the high SDI and high-middle SDI regions but increased in the middle SDI, low-middle SDI, and low SDI regions. High LDL-C mainly contributed to ischemic heart diseases.

**Conclusion:**

High LDL-C contributed considerably to health burden worldwide. Males suffered worse health outcomes attributed to high LDL-C when compared to females. The burden attributed to high LDL-C increased with age. Lower SDI regions and countries experienced more health problem challenges attributed to high LDL-C as the result of social development and this should be reflected in policymaking.

## Introduction

Low-density lipoprotein cholesterol (LDL-C) is a risk factor not only considered to be the trigger of the pathogenesis of atherosclerosis (1), but also considered to play a central role in the progress and retention of arteriosclerotic cardiovascular disease (ASCVD) (2–5). The higher the level of individual LDL-C and the longer the exposure time to abnormal LDL-C plasma concentration, the higher the risk of ASCVD. On the contrary, the lower the LDL- C and the longer the time of maintaining the low level of LDL-C, the lower the incidence of an ASCVD event (6). Thus, many studies emphasized the importance of intensive lipid-lowering treatment in very high-risk patients (7–12). Studies suggested that the patients who maintained very low LDL-C levels (<50 mg/dl) during their lifetime had a lower risk for major cardiovascular events than those who maintained moderately low levels (13, 14). As to the safety, no research showed that intensive lipid lowering treatment (even the extremely low level of <30 mg/dl) would induce a higher incidence of adverse outcomes (15–18).

In Global Burden of Diseases, Injuries, and Risk Factors (GBD) studies, LDL-C ranked highly among the risk factor clusters and was ranked in the top three leading risk factors contributing to cardiovascular disease in 2019 (19). To our acknowledge, Global Burden of Diseases, Injuries, and Risk Factors Studies are the most authoritative and comprehensive effort to reveal burdens with standardized methods across a wide set of risk factors (20–25). In this work, we use the Global Burden of Diseases data from 1990 to 2019 to give an overview of LDL-C by measures such as deaths, disability-adjusted life-years (DALYs), summary exposure value (SEV), average annual percentage change (AAPC), and observed to expected ratios (O/E ratios). In this work, we discussed the reasons for disparity and indicate the existing weak points that could help governments, domestic, and international organizations identify new priorities and formulate new health strategies.

## Methods

### Data source and definitions

This study was a secondary analysis based on the data from GBD Results Tool (26).

We searched GBD Results Tool using risk factor “High LDL cholesterol” combined with variables “year” (from 1990 to 2019), “location” (global, 26 regions, 204 countries, and territories), “age” (all ages, age- standardized, age range 25–29, age range 30–34, age range 35–39, age range 40–44, age range 45–49, age range 50–54, age range 55–59, age range 60–64, age range 65–69, age range 70–74, age range 75–79, age range 80–84, age range 85–89, age range 90–94, age range 95 plus), “sex” (both sex, male, female), and “metric” (number, rate). The data were quantified and diagrammed to show detailed health situations related to high LDL-C.

The outcome was demonstrated with (1) deaths: Deaths occurring in a population during a certain time period; (2) DALYs: The sum of years lost due to premature death (YLLs) and years lived with disability (YLDs). DALYs are also defined as years of healthy life lost; (3) Summary exposure value (SEV): SEV is the relative risk-weighted prevalence of exposure, setting the scale from 0% when there is no excess risk for a population to 100% when the population is at the highest level of risk. A decline in SEV indicates reduced exposure to a given risk factor and an increase in SEV indicates increased exposure; (4) O/E ratio of Deaths or DALYs: the ratio of observed to expected Deaths or DALYs attributed to high LDL-C based on Socio-demographic Index. It demonstrates high LDL-C for countries that are overperforming or underperforming related to their level of development. An O/E ratio of 1.0 indicates that observed Deaths or DALYs level equals our expectation. O/E ratio less than 1.0 indicates that observed Deaths or DALYs level is better than expected. O/E ratio greater than 1.0 indicates that observed Deaths or DALYs level is worse than expected. O/E ratio provides a benchmarking tool to help to focus local decision making; and (5) average annual percentage change (AAPC): Average annual percentage change is a simple mathematical concept that represents the degree of change over time (25).

Socio-demographic Index is a composite indicator of overall development status that was originally constructed in GBD 2015 and has been used from then on. SDI is derived from components that correlate strongly with health outcomes. It consists of (1) the total fertility rate among women younger than 25 years, (2) mean education for those aged 15 years or older, and (3) lagged distributed income per capita. The resulting metric ranges from 0 to 1, with higher values implying higher levels of socioeconomic development. Countries were divided into five quintiles according to SDI in GBD 2019: high SDI (0.805129–1), high middle SDI (0.689504–0.805129), middle SDI (0.607679–0.689504), low middle SDI (0.454743–0.607679), and low SDI (0–0.454743) (27).

### Statistical analysis

The GBD 2019 used the comparative risk assessment approach to quantify the associations between risk factors and outcomes (20). Data are expressed as the value with a 95% confidence interval (CI). Age-standardized rates of deaths and DALYs are expressed as the number per 100,000 persons. The Joinpoint Regression Program 4.2.0.1 was used to analyze the average annual percentage change. Associations of ASDR, age-standardized DALYs rate, and age-standardized SEVs rate with SDI were tested via linear regression analysis by STATA 15.1 Special Edition License (Stata Corporation, College Station, TX, USA, serial number: 401506209499). A P value <0.05 was considered statistically significant.

## Result

### Global burden attributed to high LDL cholesterol by year

**Figure 1:**
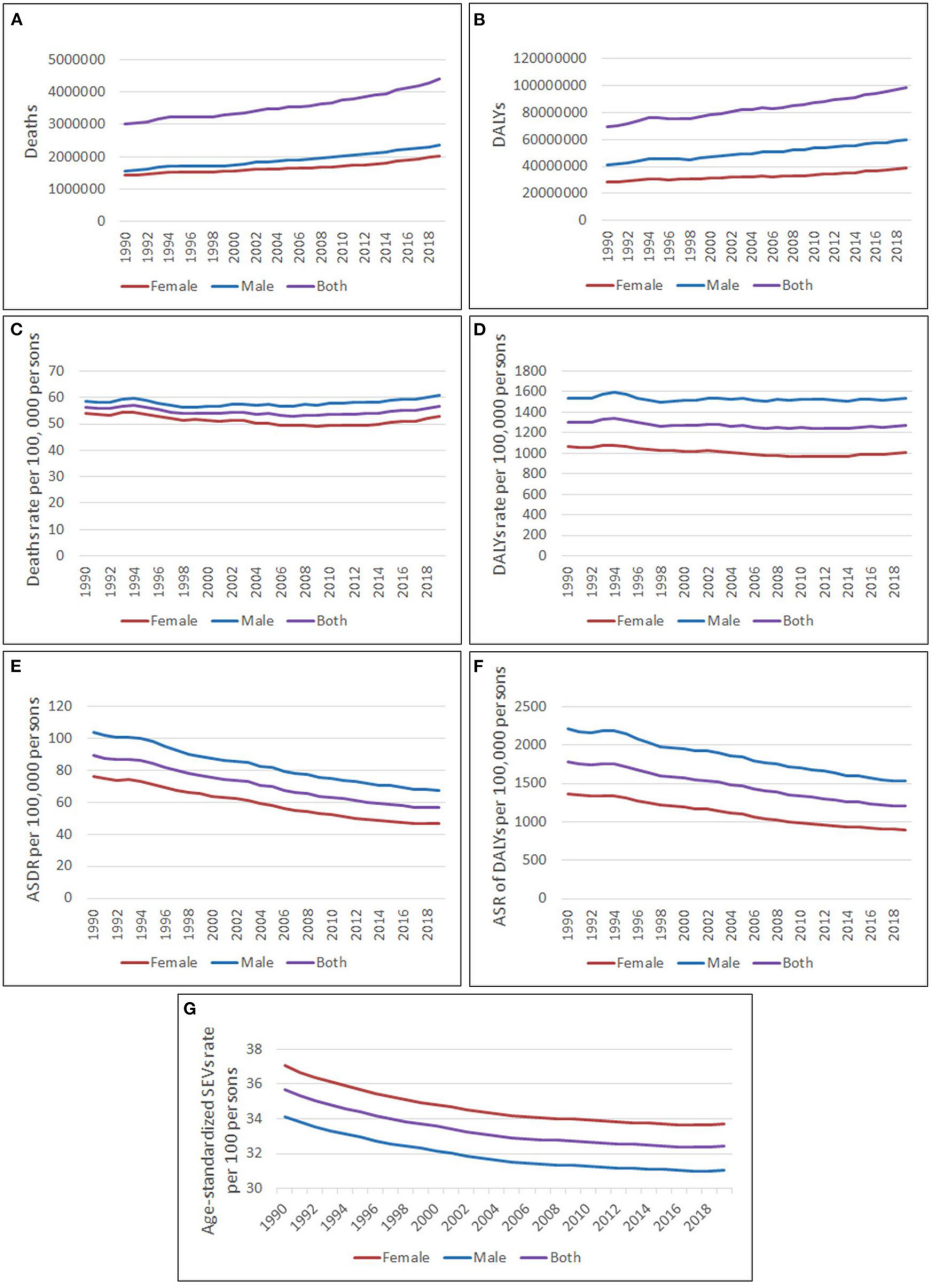
Global burden attributed to high LDL-C from 1990 to 2019. **(A)** Deaths number; **(B)** DALYs number; **(C)** Deaths rate per 100, 000 persons; **(D)** DALYs rate per 100,000 persons; **(E)** Age-standardized deaths rate per 100,000 persons; **(F)** Age-standardized DALYs rate per 100,000 persons; **(G)** Age-standardized SEVs rate per 100 persons.

Globally, 4.4 million (95% CI, 3.3–5.7) deaths were attributed to high LDL-C in 2019, which accounted for 12.6% of all risk-related deaths and had risen by 46.7% compared to 1990. In 2019, 98.6 million (95% CI, 80.3–119.0) DALYs were attributed to high LDL-C, which accounted for 81.3% of all risk-related DALYs and had increased by 41.5% compared to 1990 (Figure 1).

The deaths rate attributed to high LDL-C was 56.8 per 100,000 persons (95% CI, 42.7–73.0) in 2019, which had increased by 1.2% compared to 1990. The DALYs rate attributed to high LDL-C was 1,274.6 per 100,000 persons (95% CI, 1,038.3–1,537.8) in 2019, which had decreased by 2.2% compared to 1990. Both deaths rate and DALYs rate changed mildly during the years from 1990 to 2019 (Figure 1).

The age-standardized deaths rate per 100,000 persons had decreased by 36.7% from 89.3 (95% CI, 67.1–115.6) in 1990 to 56.5 (95% CI, 41.8–73.6) in 2019. The age-standardized DALYs rate per 100,000 persons had decreased by 32.2% from 1,779.9 per 100,000 persons (95% CI, 1,465.1–2,154.3) in 1990 to 1,207.2 per 100,000 persons (95% CI, 975.1–1,461.1) in 2019 (Figure 1).

The age-standardized SEVs rate had decreased from 35.7% (95% CI, 32.9%−38.7%) in 1990 to 32.4% (95% CI, 29.5%−35.6%) in 2019 (Figure 1).

### Global burden attributed to high LDL cholesterol by gender and age

The line chart showed that males had a similar trend to females. However, the data indicated a greater impact on males than on females. The number, rate, age standardized rate of deaths, and DALYs attributed to high LDL-C were higher in males than in females from 1990 to 2019. In 2019, the ASDR per 100,000 persons was 67.3 (95% CI, 50.8–86.4) in males and 46.5 (95% CI, 32.7–62.4) in females. The age-standardized DALYs rate per 100,000 persons was 1,528.7 (95% CI, 1,250.3–1,833.4) in males and 898.3 (95% CI, 706.0–1,120.4) in females in 2019. However, the age-standardized SEVs rate was 31.1% (95% CI, 28.1%-34.3%) in males, which was lower than 33.7% (95% CI, 30.8%-36.9%) in females (Figure 1). The average annual percentage change (AAPC) of ASDR, age-standardized DALYs rate, and age-standardized SEVs rate in males were −1.56, −1.36, and −0.31%, while they were −1.86, −1.62, and −0.30% in females.

**Figure 2:**
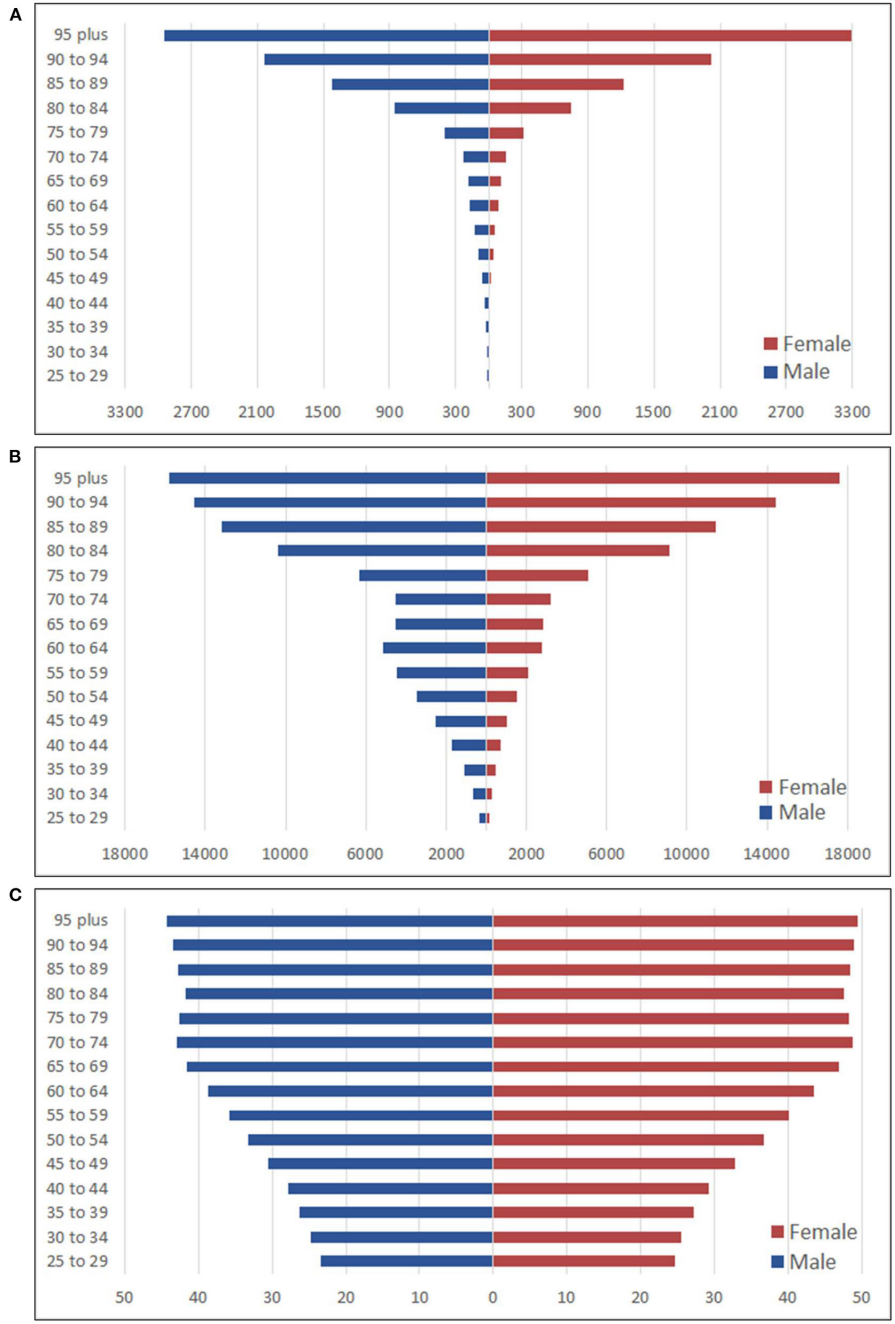
Burden of different age group in males and females attributed to high LDL-C. **(A)** Deaths rate per 100,000 persons; **(B)** DALYs rate per 100,000 persons; **(C)** SEV rate per 100 persons.

In 2019, the deaths rate per 100,000 persons increased as the age grew in both genders. Especially when reaching the age of 80, the increases were remarkable and peaked at the age group 95 plus. Both sexes shared similar trends in different age groups. The deaths rate per 100,000 persons in males was always higher than that in females from the same age group, except for the age group 95 plus when females finally outpaced males and reached the peak at 3,288.9 per 100,000 persons. When it came to the DALYs rate per 100,000 persons, it increased with age in both sexes except for the age group 60–64 males. Also, the DALYs rate per 100,000 persons in females was always lower than that in males until the age group 95 plus, where females surpassed the counterparty and reached the peak at 17,606.1 per 100,000 persons (Figure 2).

The SEVs rate of females was higher than that of males in different age groups in 2019. The highest SEVs rates for both males and females were in the 95 plus age group. The age group 90–94 was the second-highest, followed by the age group 70–74 in third for both sexes. Overall, the SEVs rate increased as the age increased (Figure 2).

### Global burden attributed to high LDL cholesterol by regions

**Figure 3:**
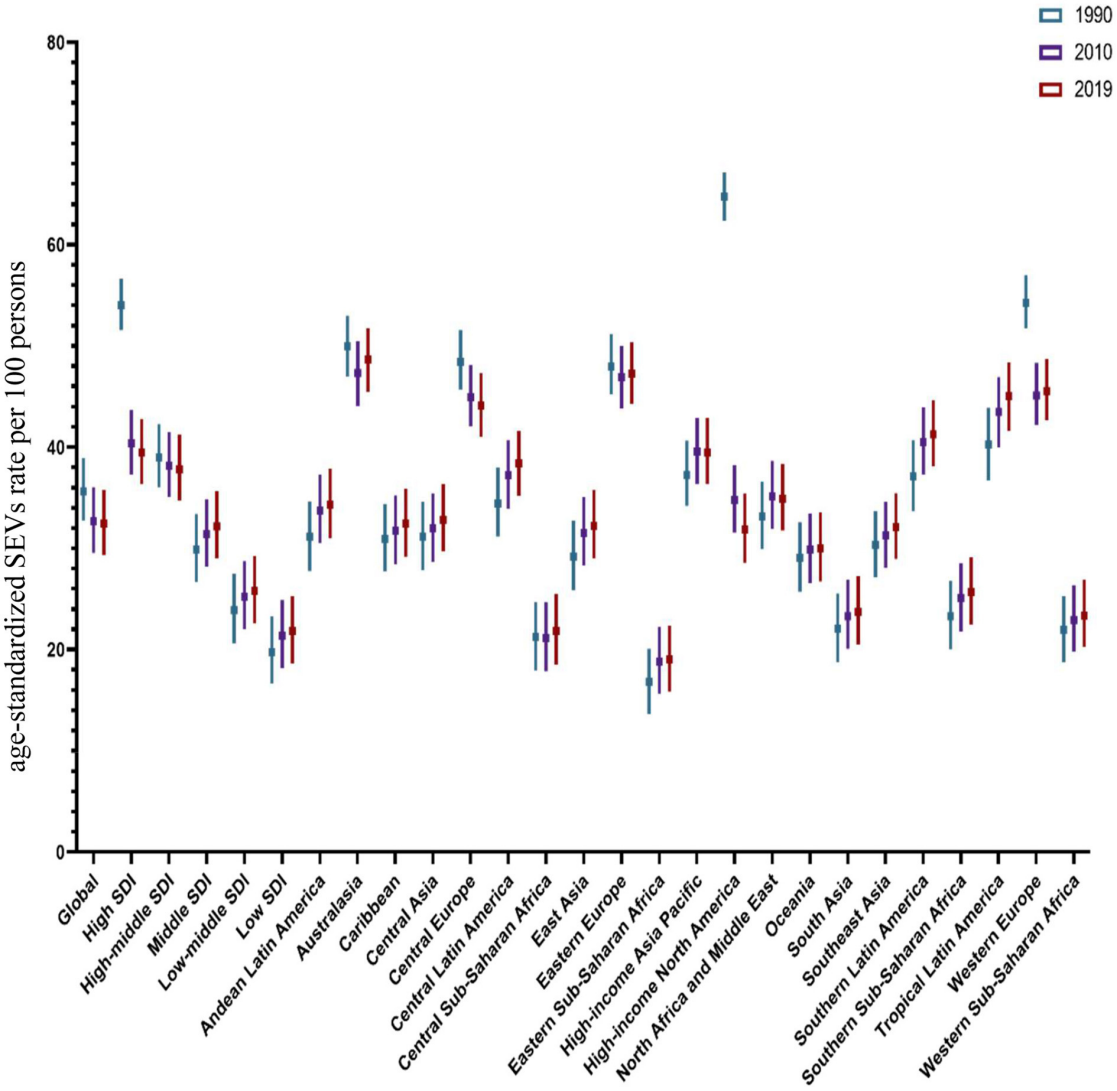
The age-standardized SEVs rate attributed to high LDL-C in 1990–2010–2019.

Among the 21 regions included in GBD, regarding the age-standardized SEVs rate per 100 persons, High-income North America experienced a dramatic drop with a value of 50.7% during 1990–2019. Eastern Sub-Saharan Africa, which increased by 13.1% during 1990–2019, remained the lowest in 2019. Australasia, Eastern Europe, and Western Europe, which experienced decreases from 1990 to 2019, were still among the high-ranked regions. Oceania, which increased slightly from 1990 to 2019, was among the low-ranked regions. Central Asia, Andean Latin America, North Africa and the Middle East, and High-income Asia Pacific placed in the median level and increased mildly from 1990 to 2019 (Figure 3).

**Figure 4:**
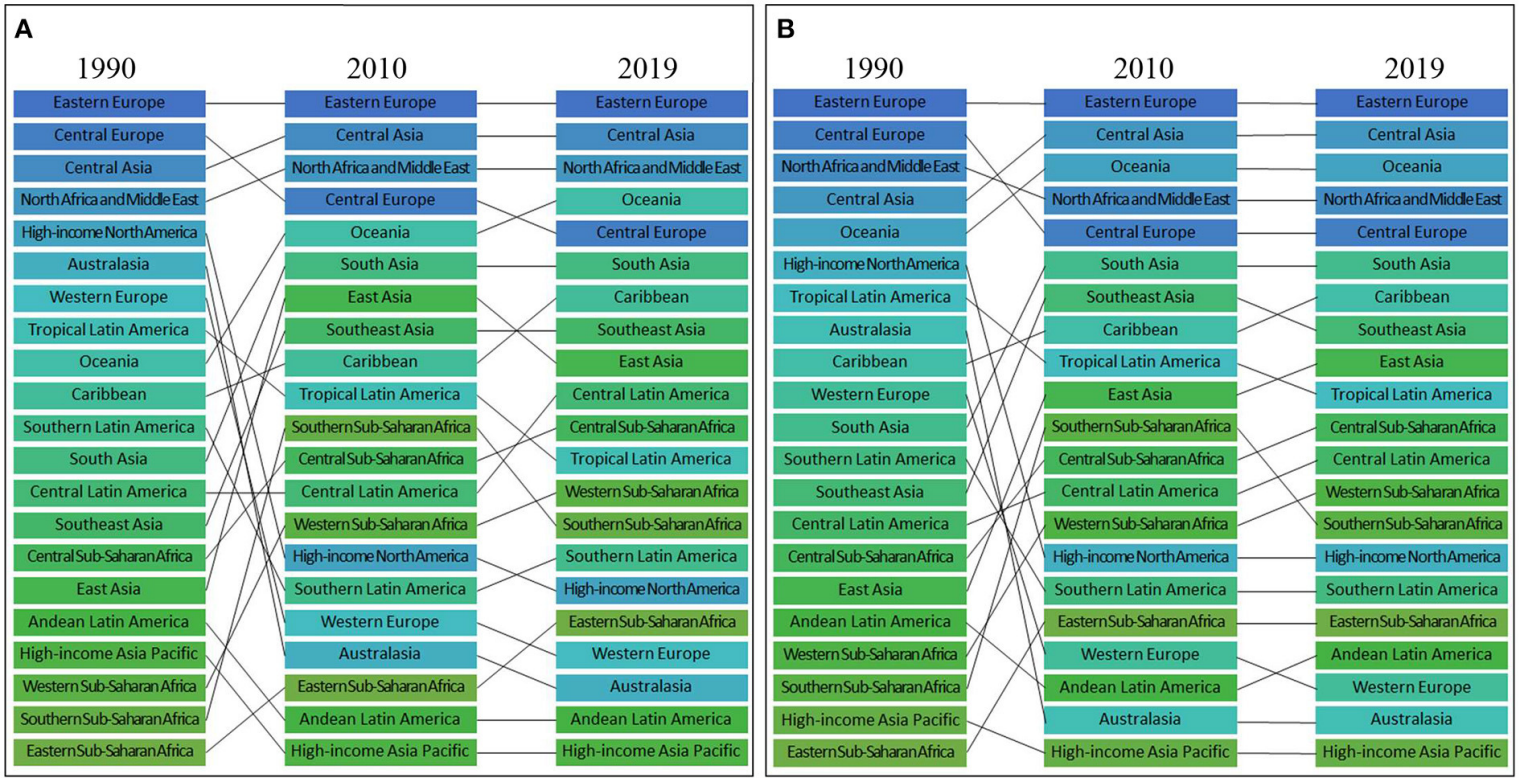
Ranks of 21 regions according to ASDR **(A)** and age-standardized DALYs rate **(B)** attributed to high LDL-C in 1990–2010–2019.

Among the 21 regions, regarding the highest ASDR per 100,000 attributed to high LDL-C, Eastern Europe remained the highest from 1990 to 2019 with a total decline of −17.6%. Central Asia was the second-highest region in 2019 with a total increase of 10.0% from 1990 to 2019. North Africa and the Middle East was the third-highest region in 2019 with a decline of −26.5% from 1990 to 2019 (Figure 4) and (Tables 1, 2).

**Table 1:** The age-standardized deaths rate (ASDR) and its changes attributed to high LDL-C in 1990–2010–2019.

**Location**	**ASDR** **(per 100,000 persons)** **in 1990**	**ASDR** **(per 100,000 persons)** **in 2010**	**ASDR** **(per 100,000 persons)** **in 2019**	**ASDR change from 1990 to 2010**	**ASDR change from 2010 to 2019**	**ASDR change from 1990 to 2019**
Global	89.3 (67.1, 115.6)	63.2 (47.2, 81.9)	56.5 (41.8, 73.6)	−29.3% (−31.5, −26.8%)	−10.6% (−14.9, −6.5%)	−36.7% (−40.6, −33.1%)
**Socio-demographic index**						
High SDI	93.9 (70.9, 119.7)	38.0 (27.8, 49.6)	32.9 (24.0, 43.5)	−59.6% (−61.7, −57.3%)	−13.3% (−15.5, −11.2%)	−64.9% (−67.1, −62.8%)
High-middle SDI	115.8 (86.9, 151.9)	87.5 (65.1, 114.7)	70.7 (51.8, 93.7)	−24.4% (−26.7, −21.9%)	−19.2% (−24.0, −14.9%)	−38.9% (−42.8, −35.5%)
Middle SDI	66.5 (49.8, 87.2)	67.0 (49.2, 88.2)	62.6 (45.5, 82.5)	0.7% (−5.6, 6.3%)	−6.6% (−13.0, 0.1%)	−5.9% (−15.0, 2.2%)
Low–middle SDI	60.4 (45.4, 79.1)	59.0 (44.2, 76.7)	58.3 (43.1, 76.1)	−2.3% (−8.5, 4.4%)	−1.1% (−8.8, 7.4%)	−3.3% (−13.7, 6.1%)
Low SDI	51.9 (37.9, 70.5)	51.8 (38.1, 69.0)	49.8 (36.0, 66.4)	−0.3% (−10.3, 8.1%)	−3.7% (−9.9, 2.9%)	−3.9% (−16.1, 6.9%)
**Region**						
Andean Latin America	47.9 (35.1, 64.1)	31.8 (23.4, 42.7)	29.4 (20.5, 40.2)	−33.6% (−41.1, −24.3%)	−7.6% (−23.4, 9.2%)	−38.6% (−50.8, −24.8%)
Australasia	96.0 (71.4, 122.8)	36.9 (26.0, 49.6)	31.4 (22.1, 42.0)	−61.5% (−64.9, −58.7%)	−15.0% (−17.9, −12.2%)	−67.3% (−70.4, −64.5%)
Caribbean	81.7 (61.0, 105.6)	56.2 (41.5, 73.2)	56.9 (41.2, 74.5)	−31.2% (−36.2, −25.7%)	1.2% (−10.6, 15.2%)	−30.4% (−39.0, −21.1%)
Central Asia	141.7 (105.8, 182.0)	181.9 (134.1, 235.0)	155.8 (111.2, 206.2)	28.4% (23.9, 33.2%)	−14.4% (−20.9, −7.5%)	10.0% (1.1, 19.2%)
Central Europe	168.2 (126.8, 215.3)	103.9 (76.2, 137.8)	86.0 (60.3, 116.3)	−38.2% (−41.2, −35.7%)	−17.2% (−26.3, −8.3%)	−48.9% (−55.1, −43.0%)
Central Latin America	59.9 (44.4, 77.1)	45.9 (33.8, 59.6)	45.3 (32.9, 59.6)	−23.3% (−26.3, −20.9%)	−1.4% (−12.6, 11.6%)	−24.4% (−33.3, −13.7%)
Central Sub-Saharan Africa	49.5 (33.9, 69.0)	46.4 (31.6, 66.6)	45.3 (28.9, 66.9)	−6.3% (−19.7, 9.5%)	−2.5% (−16.0, 12.1%)	−8.6% (−26.3, 13.2%)
East Asia	49.0 (35.3, 68.3)	60.2 (42.3, 83.1)	55.1 (37.3, 76.4)	22.7% (7.5, 39.7%)	−8.5% (−20.3, 4.4%)	12.3% (−5.6, 32.3%)
Eastern Europe	191.0 (144.7, 248.4)	198.7 (152.4, 252.1)	157.4 (117.0, 203.2)	4.1% (−0.8, 9.3%)	−20.8% (−27.3, −14.1%)	−17.6% (−24.9, −9.7%)
Eastern Sub-Saharan Africa	32.2 (22.2, 45.9)	33.9 (22.6, 47.6)	33.2 (21.4, 47.4)	5.4% (−12.1, 21.0%)	−2.1% (−10.5, 5.2%)	3.2% (−20.0, 24.5%)
High-income Asia Pacific	47.3 (33.1, 66.6)	20.4 (14.4, 28.5)	16.7 (11.6, 23.8)	−56.7% (−60.2, −53.8%)	−18.3% (−20.9, −15.9%)	−64.6% (−67.9, −61.9%)
High-income North America	105.8 (81.4, 131.7)	41.6 (30.5, 54.1)	37.2 (27.1, 49.0)	−60.7% (−63.7, −57.6%)	−10.7% (−13.1, −8.7%)	−64.9% (−68.0, −61.9%)
North Africa and the Middle East	140.5 (107.6, 178.2)	111.5 (83.4, 143.0)	103.3 (75.3, 135.6)	−20.7% (−26.3, −15.8%)	−7.3% (−13.6, −0.8%)	−26.5% (−35.2, −18.6%)
Oceania	84.5 (63.6, 112.6)	92.0 (70.3, 124.0)	91.1 (67.2, 123.5)	8.8% (−2.2, 22.7%)	−1.0% (−11.8, 10.2%)	7.7% (−9.6, 30.0%)
South Asia	62.7 (47.5, 83.1)	61.7 (46.6, 78.7)	59.9 (44.5, 77.7)	−1.6% (−10.0, 8.4%)	−2.9% (−14.5, 9.3%)	−4.4% (−19.4, 9.4%)
Southeast Asia	56.2 (42.1, 75.2)	57.9 (43.6, 76.8)	55.2 (40.2, 73.0)	3.0% (−4.5, 11.2%)	−4.7% (−13.3, 3.4%)	−1.8% (−14.0, 9.4%)
Southern Latin America	78.0 (58.0, 101.2)	41.5 (31.1, 53.5)	37.3 (27.8, 48.5)	−46.8% (−48.8, −44.7%)	−10.2% (−13.8, −6.5%)	−52.2% (−54.8, −49.5%)
Southern Sub-Saharan Africa	38.5 (27.9, 52.9)	50.6 (37.1, 67.8)	41.6 (29.5, 57.5)	31.3% (20.5, 45.9%)	−17.8% (−24.1, −12.6%)	7.9% (−2.5, 18.6%)
Tropical Latin America	87.7 (67.1, 113.7)	51.2 (39.6, 65.8)	43.3 (33.5, 56.0)	−41.6% (−43.9, −39.2%)	−15.5% (−18.4, −12.2%)	−50.6% (−53.2, −48.0%)
Western Europe	91.8 (69.2, 118.8)	37.5 (27.0, 50.1)	32.2 (23.0, 43.4)	−59.1% (−61.6, −56.6%)	−14.1% (−15.9, −12.5%)	−64.9% (−67.5, −62.3%)
Western Sub-Saharan Africa	46.5 (30.9, 67.1)	44.1 (30.2, 60.8)	43.0 (29.1, 59.4)	−5.2% (−28.6, 12.4%)	−2.4% (−13.0, 9.0%)	−7.5% (−30.0, 10.9%)

**Table 2:** The age-standardized DALYs rate (ASR of DALYs) and its changes attributed to high LDL-C in 1990–2010–2019.

**Location**	**ASR of DALYs** **(per 100,000 persons)** **in 1990**	**ASR of DALYs** **(per 100,000 persons)** **in 2010**	**ASR of DALYs** **(per 100,000 persons)** **in 2019**	**ASR of DALYs change from 1990 to 2010**	**ASR of DALYs change from 2010 to 2019**	**ASR of DALYs change from 1990 to 2019**
Global	1,779.9 (1,465.1, 2,154.3)	1,342.8 (1,103.8, 1,628.1)	1,207.1 (975.1, 1,461.1)	−24.6% (−27.2, −21.6%)	−10.1% (−14.7, −5.4%)	−32.2% (−36.7, −27.8%)
**Socio-demographic index**						
High SDI	1,777.6 (1,475.5, 2,109.5)	767.1 (633.0, 919.1)	673.6 (551.6, 813.6)	−56.8% (−58.5, −55.1%)	−12.2% (−15.0, −9.5%)	−62.1% (−64.1, −59.9%)
High-middle SDI	2,249.0 (1,860.7, 2,742.3)	1,725.7 (1,412.8, 2,080.2)	1,372.1 (1,107.4, 1,671.7)	−23.3% (−25.8, −19.9%)	−20.5% (−25.5, −15.7%)	−39.0% (−43.1, −35.1%)
Middle SDI	1,451.8 (1,183.6, 1,785.0)	1,407.7 (1,140.6, 1,725.8)	1,318.0 (1,060.4, 1,606.7)	−3.0% (−9.0, 2.6%)	−6.4% (−13.0, 0.6%)	−9.2% (−17.9, −1.2%)
Low-middle SDI	1,426.0 (1,150.3, 1,759.6)	1,394.0 (1,127.5, 1,707.9)	1,367.5 (1,088.8, 1,676.5)	−2.2% (−9.1, 5.5%)	−1.9% (−10.2, 7.3%)	−4.1% (−15.0, 6.6%)
Low SDI	1,231.4 (959.0, 1,564.7)	1,218.7 (956.9, 1,525.9)	1,166.2 (909.0, 1,456.0)	−1.0% (−10.3, 8.2%)	−4.3% (−11.5, 3.3%)	−5.3% (−16.7, 6.2%)
**Region**						
Andean Latin America	990.4 (791.3, 1,226.3)	658.8 (537.7, 814.2)	592.6 (448.6, 768.5)	−33.5% (−41.3, −24.1%)	−10.0% (−25.7, 7.3%)	−40.2% (−52.1, −26.1%)
Australasia	1,802.7 (1,486.7, 2,147.1)	648.6 (523.9, 788.5)	550.0 (441.6, 669.3)	−64.0% (−66.0, −62.0%)	−15.2% (−18.0, −12.6%)	−69.5% (−71.4, −67.3%)
Caribbean	1,740.1 (1,415.5, 2,093.8)	1,226.9 (978.8, 1,501.9)	1,268.5 (989.1, 1,582.9)	−29.5% (−35.5, −22.8%)	3.4% (−9.8, 19.8%)	−27.1% (−37.6, −16.1%)
Central Asia	2,958.8 (2,409.9, 3,553.8)	3,632.4 (2,932.8, 4,377.8)	3,067.5 (2,409.3, 3,821.8)	22.8% (18.1, 27.7%)	−15.6% (−22.9, −7.4%)	3.7% (−5.6, 14.6%)
Central Europe	3,275.1 (2,728.9, 3,895.7)	1,906.2 (1,552.1, 2,313.7)	1,549.6 (1,199.8, 1,947.4)	−41.8% (−43.8, −39.6%)	−18.7% (−28.3, −8.8%)	−52.7% (−58.7, −46.9%)
Central Latin America	1,235.7 (1,012.7, 1,482.5)	936.8 (769.9, 1,117.9)	924.1 (727.0, 1,141.8)	−24.2% (−26.5, −22.2%)	−1.4% (−13.6, 12.7%)	−25.2% (−34.6, −13.8%)
Central Sub-Saharan Africa	1,122.4 (814.7, 1,507.9)	1,018.5 (737.4, 1,351.1)	976.6 (675.2, 1,350.1)	−9.3% (−23.3, 7.9%)	−4.1% (−18.8, 12.2%)	−13.0% (−31.4, 9.9%)
East Asia	1,050.7 (831.8, 1,349.4)	1,129.6 (883.0, 1,422.1)	1,048.1 (801.9, 1,338.9)	7.5% (−5.7, 23.2%)	−7.2% (−19.6, 6.9%)	−0.3% (−16.2, 18.8%)
Eastern Europe	3,674.0 (3,038.5, 4,433.0)	4,011.9 (3,340.5, 4,769.2)	3,079.1 (2,456.4, 3,744.3)	9.2% (4.0, 16.8%)	−23.3% (−30.6, −15.9%)	−16.2% (−24.5, −6.7%)
Eastern Sub-Saharan Africa	736.3 (546.3, 976.2)	748.7 (548.9, 985.8)	718.0 (511.8, 955.1)	1.7% (−15.5, 18.4%)	−4.1% (−12.3, 3.9%)	−2.5% (−25.1, 19.6%)
High-income Asia Pacific	865.6 (689.5, 1,111.9)	423.1 (343.1, 531.5)	343.2 (273.8, 434.2)	−51.1% (−53.5, −48.5%)	−18.9% (−21.5, −16.6%)	−60.4% (−62.4, −57.8%)
High-income North America	2,087.8 (1,762.9, 2,419.9)	874.0 (718.5, 1,045.3)	780.3 (636.3, 938.8)	−58.1% (−60.5, −55.8%)	−10.7% (−13.4, −8.7%)	−62.6% (−65.2, −60.3%)
North Africa and the Middle East	3,201.1 (2,628.8, 3,822.8)	2,433.1 (1,987.8, 2,917.6)	2,235.4 (1,767.8, 2,767.8)	−24.0% (−29.6, −19.2%)	−8.1% (−15.2, −0.3%)	−30.2% (−39.0, −20.9%)
Oceania	2,273.2 (1,758.2, 2,950.4)	2,458.7 (1,932.6, 3,197.9)	2,396.0 (1,837.2, 3,185.8)	8.2% (−4.4, 24.1%)	−2.5% (−13.7, 10.2%)	5.4% (−13.0, 29.5%)
South Asia	1,541.5 (1,234.0, 1,911.5)	1,537.4 (1,243.7, 1,858.1)	1,466.4 (1,154.4, 1,816.8)	−0.3% (−9.3, 10.7%)	−4.6% (−16.2, 8.2%)	−4.9% (−20.0, 9.7%)
Southeast Asia	1,282.3 (1,029.8, 1,606.5)	1,310.2 (1,067.1, 1,626.3)	1,249.2 (996.3, 1,543.5)	2.2% (−6.0, 11.2%)	−4.7% (−13.7, 4.3%)	−2.6% (−14.4, 9.7%)
Southern Latin America	1,510.5 (1,236.6, 1,819.5)	831.9 (691.5, 987.3)	733.4 (606.9, 885.5)	−44.9% (−46.8, −43.0%)	−11.8% (−15.4, −8.4%)	−51.4% (−54.0, −48.9%)
Southern Sub-Saharan Africa	898.5 (705.0, 1,143.4)	1,087.7 (866.9, 1,361.7)	865.0 (666.5, 1,092.1)	21.1% (10.8, 34.1%)	−20.5% (−27.0, −14.3%)	−3.7% (−13.4, 5.8%)
Tropical Latin America	1,921.8 (1,599.2, 2,306.4)	1,166.9 (977.3, 1,378.6)	982.7 (819.5, 1,163.9)	−39.3% (−41.5, −37.1%)	−15.8% (−18.8, −12.5%)	−48.9% (−51.4, −46.3%)
Western Europe	1,697.3 (1,408.6, 2,037.3)	677.0 (548.9, 817.0)	572.5 (462.4, 695.2)	−60.1% (−61.6, −58.5%)	−15.4% (−17.2, −14.0%)	−66.3% (−67.9, −64.7%)
Western Sub-Saharan Africa	965.5 (688.0, 1,330.3)	907.2 (668.0, 1,199.7)	875.8 (637.0, 1,151.0)	−6.0% (−27.9, 13.0%)	−3.5% (−15.9, 10.6%)	−9.3% (−30.1, 10.0%)

Regarding the age-standardized DALYs rate per 100,000 persons, Eastern Europe was the highest in both 1990 and 2019. Its overall decrease was −16.2% from 1990 to 2019. Central Asia was the second-highest region in 2019. From 1990 to 2019, the overall trend of Central Asia was ascending (3.7%). Oceania was the third-highest region in 2019, and it experienced a total increase of 5.4% from 1990 to 2019 (Figure 4) and (Tables 1, 2).

Among the low ASDR regions, in 2019, the top three consisted of High-income Asia Pacific, Andean Latin America, and Australasia. From 1990 to 2019, the change was −64.6, −38.6, and −67.3% respectively (Figure 4) and (Tables 1, 2).

Among the low age-standardized DALYs rate regions, in 2019, the top three were High-income Asia Pacific, Australasia, and Western Europe. From 1990 to 2019, the overall change was −60.4, −69.5, and −66.3% respectively (Figure 4) and (Tables 1, 2).

### Global burden attributed to high LDL cholesterol by SDI

**Figure 5:**
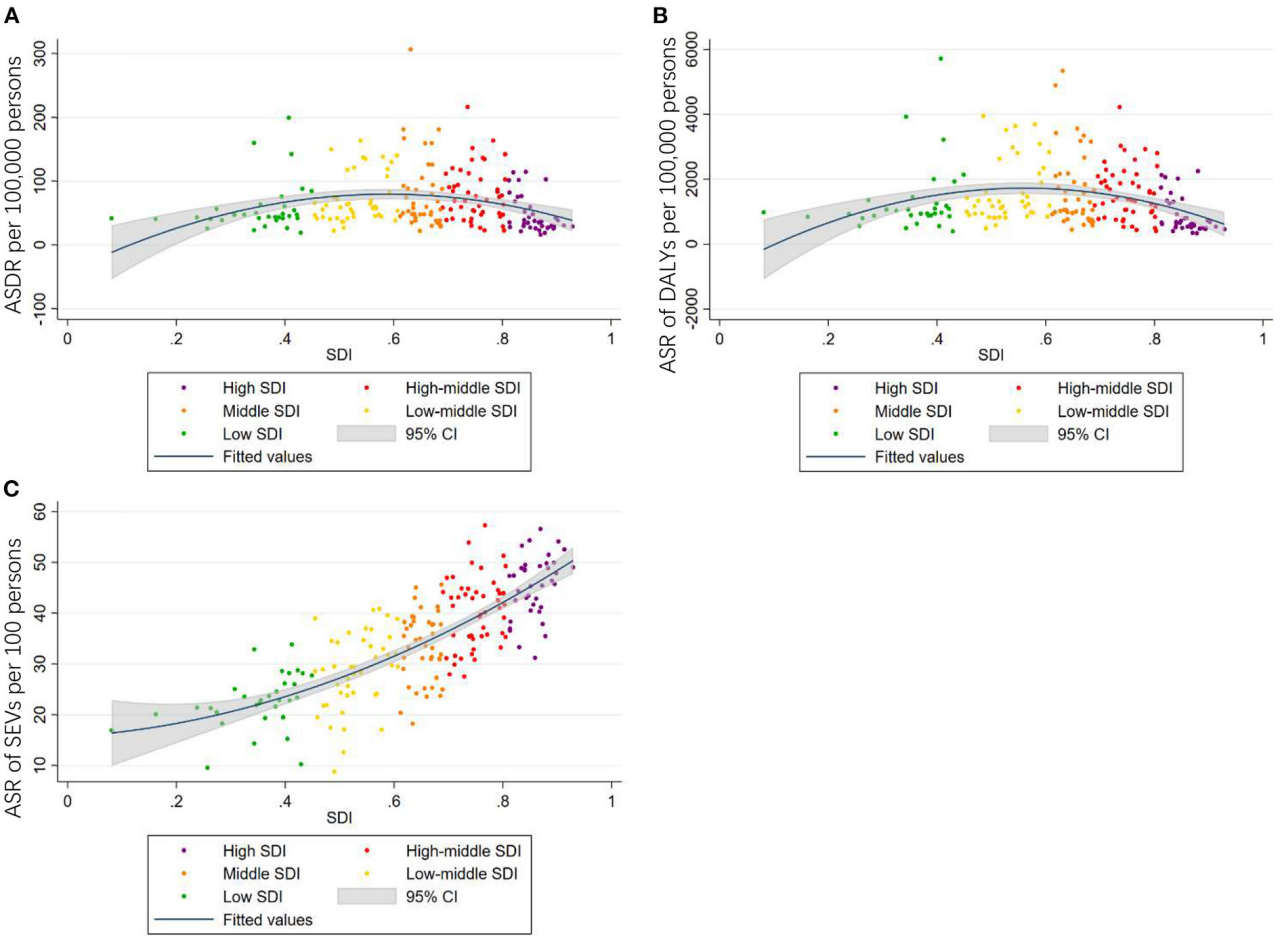
The correlations between ASDR **(A)**, age-standardized DALYs rate **(B)**, age-standardized SEVs rate **(C)**, and SDI of 204 countries and territories in 2019.

Among the five GBD SDI quintiles in 2019, high-middle SDI countries showed the highest ASDR and age-standardized DALYs rate, followed by countries with middle SDI and low-middle SDI. High SDI countries had the lowest ASDR and age-standardized DALYs rate, which was less than half compared to high-middle SDI countries. Detailed information is demonstrated in Tables 1, 2. We also analyzed the relationships between age-standardized SEVs rate and SDI among the 204 included countries and territories, which revealed positive correlations between the parameters (*r* = 0.7753, *P* < 0.001; Figure 5).

**Figure 6:**
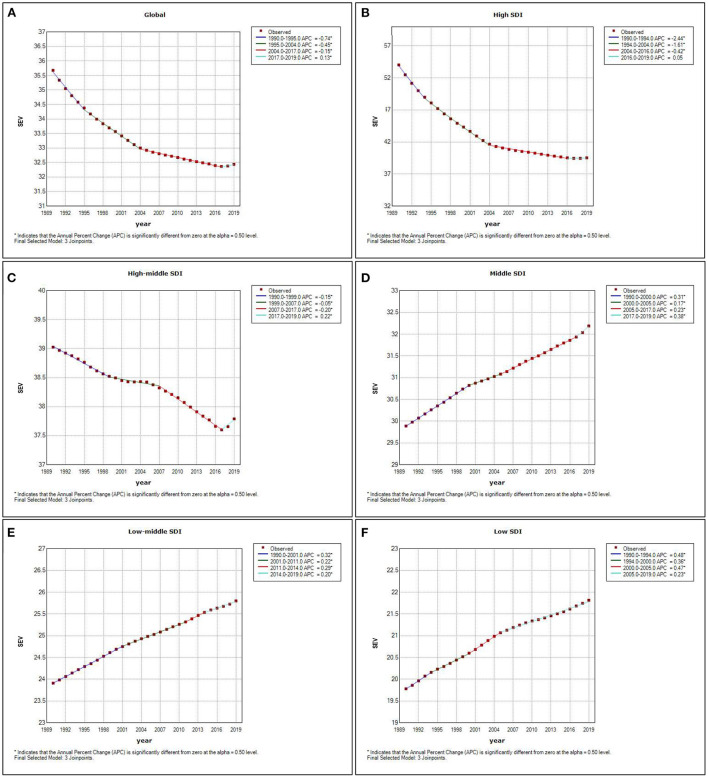
Average annual percentage change of age-standardized SEVs rate attributed to high LDL-C by year in the whole world **(A)**, high SDI regions **(B)**, high-middle SDI regions **(C)**, middle SDI regions **(D)**, low-middle SDI regions **(E)**, and low SDI regions **(F)**.

Furthermore, when analyzing the average annual percentage change (AAPC) of age-standardized SEVs rate, the high SDI and high-middle SDI regions as global showed a descending trend, while the middle SDI, low-middle SDI, and low SDI regions showed an ascending trend (Figure 6).

### Global burden attributed to high LDL cholesterol by countries

**Figure 7:**
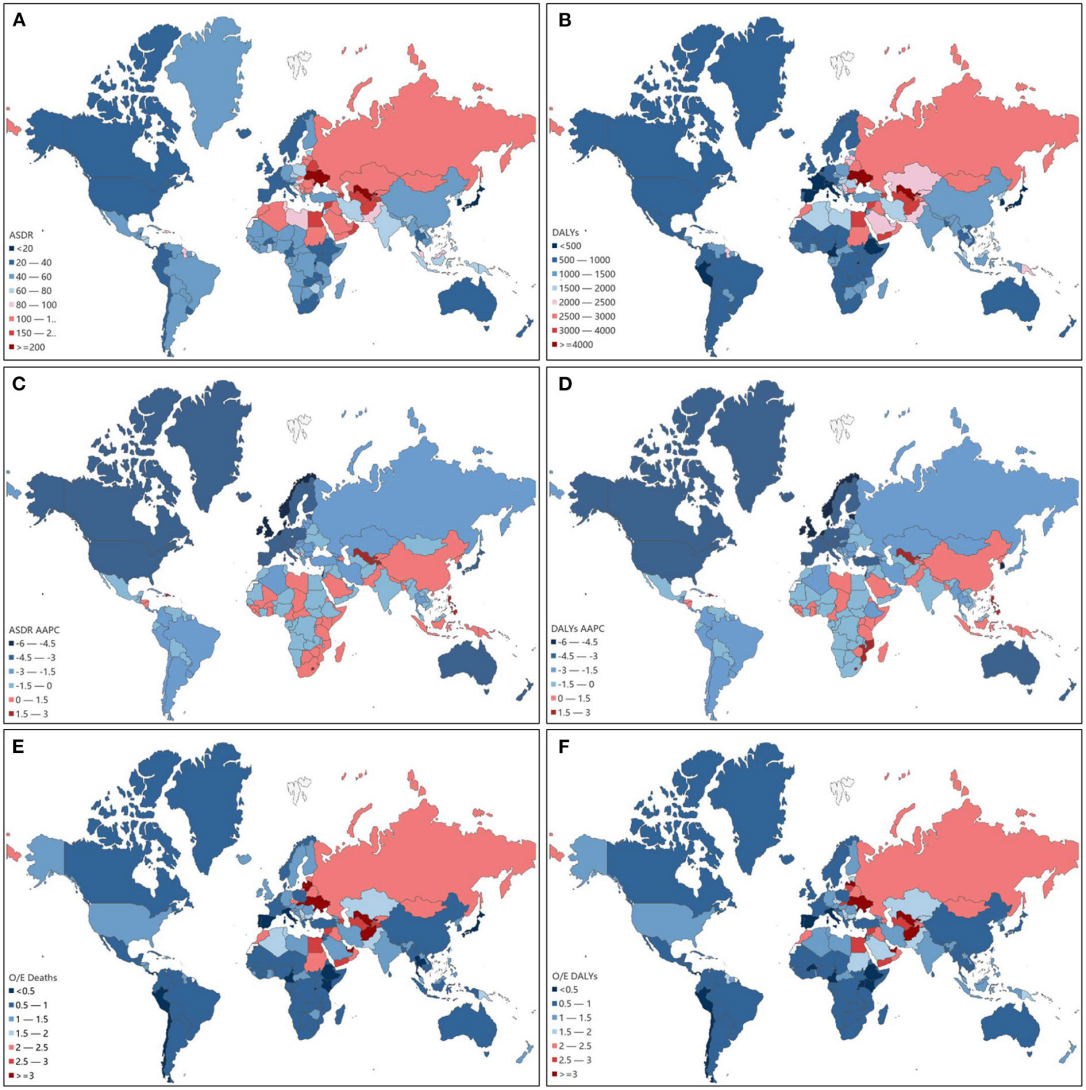
ASDR attributed to high LDL-C in 204 countries and territories in 2019 **(A)**. Age-standardized DALYs rate attributed to high LDL-C in 204 countries and territories in 2019 **(B)**. Average annual percentage change (AAPC) of ASDR attributed to high LDL-C in 204 countries and territories from 1990 to 2019 **(C)**. AAPC of age-standardized DALYs rate attributed to high LDL-C in 204 countries and territories from 1990 to 2019 **(D)**. O/E ratio of ASDR attributed to high LDL-C in 204 countries and territories in 2019 **(E)**. O/E ratio of age-standardized DALYs rate attributed to high LDL-C in 204 countries and territories in 2019 **(F)**.

The global map depicted the geographic distribution of age- standardized rates of deaths and DALYs attributed to high LDL-C in 2019. Among the 204 countries and territories worldwide, the highest ASDR per 100,000 persons attributed to high LDL-C was in Uzbekistan with a value of 306.9 (95% CI, 209.6–412.8), followed by Ukraine with 216.5 (95% CI, 159.8–284.3) and the Solomon Islands with 199.7 (95% CI, 149.4–258.9). The country with the lowest ASDR per 100,000 persons was Japan with a value of 16.4 (95% CI, 11.6–22.8). This was followed by the Republic of Korea with 19.2 (95% CI, 12.4–28.7) and Rwanda with 19.5 (95% CI, 10.3–31.8). The top three age-standardized DALYs rates per 100,000 persons were found in the Solomon Islands with a value of 5,716.5 (95% CI, 4,323.1–7,343.3), Uzbekistan with 5,339.3 (95% CI, 4,011.1–6,779.5), and Nauru with 4,893.0 (95% CI, 3,811.2–6,187.8). On the contrary, the lowest age-standardized DALYs rate per 100,000 persons was in the Republic of Korea with a value of 336.9 (95% CI, 248.1–458.9). The second-lowest was in Japan with 348.2 (95% CI, 283.5–433.2), and the third was in Rwanda with 397.9 (95% CI, 233.6–618.0; Figure 7).

From 1990 to 2019, the highest average annual percentage change (AAPC) of ASDR attributed to high LDL-C occurred in Uzbekistan (2.668%), followed by Lesotho (2.421%) and the Philippines (2.163%). While the highest AAPC of age-standardized DALYs rate attributed to high LDL-C occurred in the Philippines (2.944%), followed by Lesotho (2.679%) and Uzbekistan (2.053%). Furthermore, the largest AAPC decrease of ASDR was in Denmark (−5.488%), followed by Israel (−5.372%) and the Netherlands (−4.634%). Also, the largest AAPC decrease in age-standardized DALYs rate was in Denmark (−5.749%), followed by Israel (−5.558%) and the Netherlands (−5.030%; Figure 7).

The top three O/E ratios of ASDR based on SDI were Uzbekistan, Solomon Islands, and Lithuania with a value of 4.90, 4.01, and 3.49, respectively. The lowest three O/E ratios of ASDR occurred in Israel, Spain, and Peru with a value of 0.3252, 0.3280, and 0.3550, respectively. The top three O/E ratios of age-standardized DALYs rate based on SDI were the Solomon Islands, Uzbekistan, and Nauru with a value of 4.9018, 4.0511, and 3.7124 respectively. The lowest three O/E ratios of age-standardized DALYs rate were Israel, Spain, and Peru with a value of 0.2962, 0.3186, and 0.3384 (Figure 7).

### Causes of deaths and DALYs attributed to high LDL cholesterol

High LDL-C contributed to two causes, namely ischemic heart diseases (IHD) and stroke. In 2019, the percent of deaths and DALYs that were attributed to high LDL-C mediated by ischemic heart diseases was 86.1%, compared with that mediated by stroke (13.9%), and ischemic heart diseases accounted for a larger proportion. High LDL-C ranked second among the risk factors that contributed to ASDR of IHD and eighth among the risk factors that contributed to stroke. High LDL-C ranked second among the risk factors that contributed to age-standardized DALYs rate of IHD and seventh among the risk factors that contributed to stroke.

**Figure 8:**
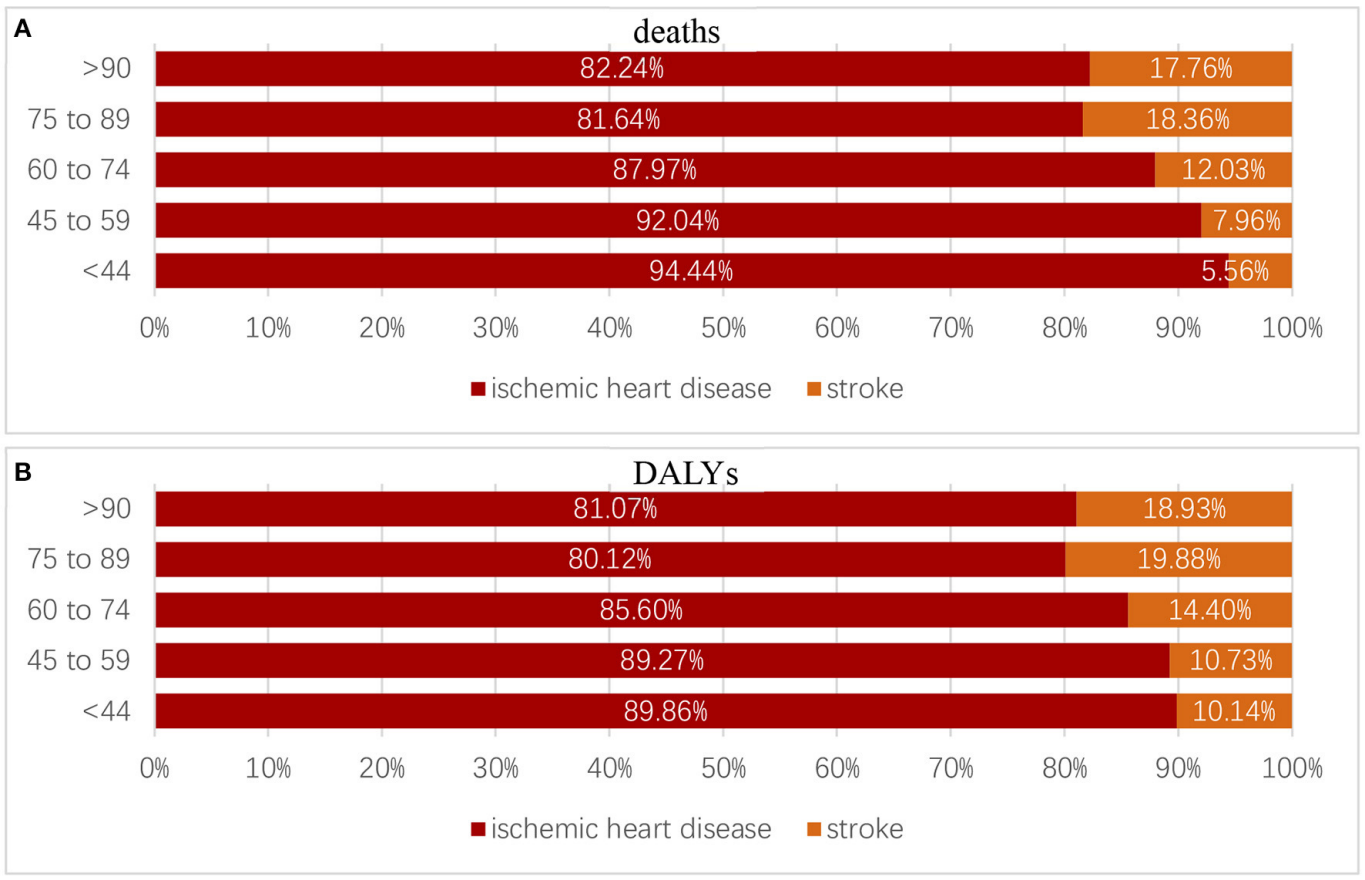
Proportion of deaths **(A)** and DALYs **(B)** caused by ischemic heart diseases and strokes in different age groups.

For different age groups, in 2019, the percentage of deaths and DALYs attributed to high LDL-C caused by ischemic heart diseases and strokes changed. According to the World Health Organization's classification of age groups, we divided the population into five groups: age group <44, age group 45–59, age group 60–74, age group 75–89, and age group >90. Both deaths and DALYs caused by IHD took dominant positions compared with that caused by strokes among different age groups but still showed a slight decrease as age grew (Figure 8).

**Figure 9:**
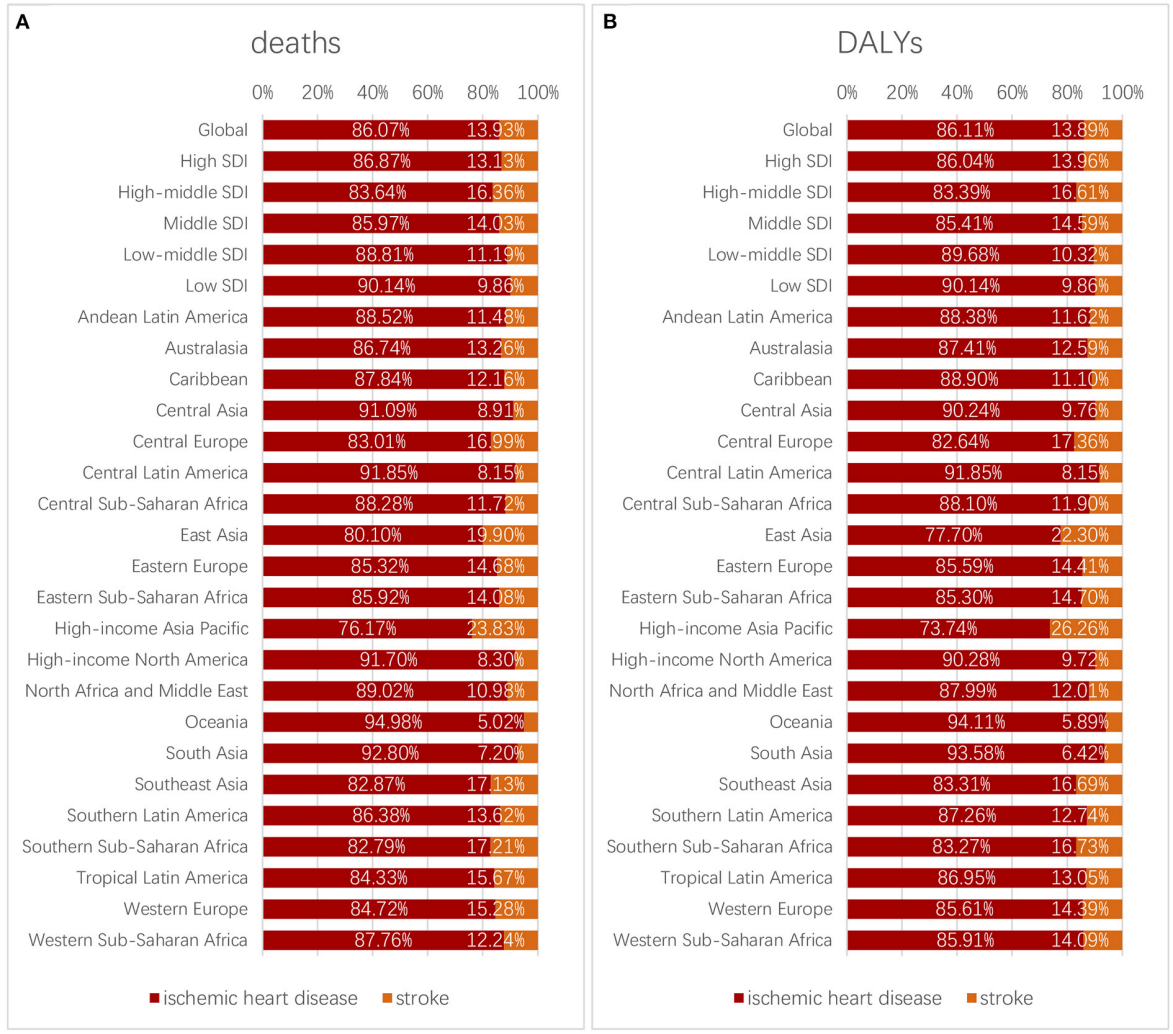
Proportion of deaths **(A)** and DALYs **(B)** caused by ischemic heart diseases and strokes in different regions.

For different regions, the top five ranked from high to low percentages of deaths caused by IHD were as follows: Oceania, South Asia, Central Latin America, High-income North America, and Central Asia. The rank of percentages of DALYs caused by IHD was the same. These places should give more attention to the issue of health detriment caused by IHD (Figure 9).

## Discussion

Globally, the deaths and DALYs numbers were increased year by year. Especially for DALYs attributed to high LDL-C, it increased by a great amount in 2019. We can infer that, if LDL-C could be improved, the burden would be reduced tremendously. The trends of both deaths and DALYs rate per 100,000 persons by year changed little. The trends of age- standardized deaths and DALYs rate descended and were consistent with the yearly change of age- standardized SEVs. Population growth and population aging may play important roles in the increased amounts of deaths and DALYs numbers, which was consistent with the data available on the United Nations website (World Population Prospects - Population Division - United Nations). As the increase of population and population aging would be inevitable in the near future, a heavier burden of high LDL-C could be foreseen. Also, it implied that, to compare the situation between different regions and populations, the measure as age-standardized deaths, DALYs, and SEVs rate would be more precise.

The impact measured by deaths and DALYs attributed to high LDL-C on males was much greater than on females in any year and for almost any age range. The conclusion was consistent with other studies (28). The SEVs rate was higher among females compared with males in the corresponding age group. It may infer that, although females suffered more exposure to high LDL-C, males still experienced worse outcomes. Few studies highlighted sex differences in cholesterol and atherosclerosis, but plenty of research focused on sex differences in longevity. A new theory was proposed that sex chromosomes may play a role in human sex differences in aging and longevity (29). And it could be conjectured that sex chromosomes may also affect the outcomes of diseases.

The highest deaths, DALYs, and SEV rates all occurred in the age group 95 plus and were strongly connected with age. It should be noted that, in both sexes, the SEV rate in the age group 70–74 ranked third among all age groups, while the deaths and DALYs rate in the age group 70–74 did not rank particularly highly. It inferred that the age group 70–74 suffered greater LDL-C exposure while the health outcome was not worse correspondingly. It could be deduced that age was a strong factor related to the impact of high LDL-C, while other factors such as diet intake, nutrition absorption, physical activity, and the application of lipids lowering drugs also played important roles in the pathology of atherosclerosis (2, 10).

In this analysis, we found that Oceania as a low-SEV country was high in both ASDR and age-standardized DALYs rate. In another study, Oceania was high in non-high-density lipoprotein cholesterol (non-HDL cholesterol) (30), which was not consistent with our study. The discrepancy may result from the measurement (non-HDL cholesterol), which was more comprehensive. Another study indicated that Oceania had a high dietary risk and highest DALYs rate of dietary-related cardiovascular disease (31). The dietary risk could affect cholesterol levels. Governments and health organizations should pay attention to the health condition of Oceania. Eastern Sub-Saharan Africa was a low-SEV region and therefore had a low ASDR and age-standardized DALYs rate, but it showed a rising trend from the years 1990 to 2019. Another study demonstrated that low- and middle-income countries had been undergoing dietary pattern changes, which shifted to energy-dense but low-nutrient foods, causing double burden of malnutrition and being overweight (32, 33). And the dietary pattern changes would exacerbate the risk of high LDL-C. This region should be alert to the changes.

Central Asia, Andean Latin America, North Africa and the Middle East, and High-income Asia Pacific were median-level-SEV countries. High-income Asia Pacific and Andean Latin America had low ASDR and age-standardized DALYs rates. While Central Asia and North Africa and the Middle East had high ASDR and age-standardized DALYs rate. Australasia, Eastern Europe, and Western Europe had high age-standardized SEVs rates in 2019. Australasia and Western Europe had low ASDR and age-standardized DALYs rates, while Eastern Europe had high ASDR and age-standardized DALYs rates. An imbalance existed between different regions. Higher exposure did not necessarily determine a worse outcome. Other risk factors may have co-influences with high LDL-C and the availability to medical care may play an important role. Notably, High-income North America and Western Europe, once considered to be heavy-burdened areas with high values of age-standardized SEVs rate, ASDR, and age-standardized DALYs rate, were less affected by high LDL-C in 2019. It implied that health policies worked effectively in these regions.

Furthermore, the scatter diagram of 204 countries showed some clues to the relationship between ASDR, age-standardized DALYs rate, age-standardized SEVs rate, and SDI. The age-standardized Deaths and DALYs rate increased together with SDI till high-middle SDI, which reached a peak then declined in high SDI. The age-standardized SEVs rate and SDI grew accordantly.

The highest age-standardized SEVs rate occurred in high SDI regions and countries. High LDL-C is a metabolic risk factor that was closely associated with diet and physical activity. It was explainable that those higher SDI regions and countries abundant in food supply and inadequate in physical activity were more likely to give rise to high LDL-C. Although the high SDI regions and countries suffered higher LDL-C exposure, due to better diagnosis and treatment conditions, their ASDR and age-standardized DALYs rate were the lowest in 2019. Combined with the average annual percentage change of age-standardized SEVs rate, the high SDI and high-middle SDI regions experienced a continuing decline. While the middle SDI, low-middle SDI, and low SDI regions were still on an upward trend. The results above indicated that, although high LDL-C as a metabolic risk factor once had been thought as a challenge mainly in high-income countries, it was now generally increased even in low-income countries. More attention should be paid to the middle SDI, low-middle SDI, and low SDI regions.

Notably, countries such as Denmark, the Netherlands, and Israel experienced significant declines of ASDR and age-standardized DALYs rate during the years from 1990 to 2019. Countries such as Uzbekistan, Lesotho, and the Philippines still had upward trends. Noticeably, Uzbekistan from the Central Asia region was among the most burdened countries with high ASDR, age-standardized DALYs rate, AAPC of deaths and DALYs, and O/E ratio. In a study, it was revealed that alcohol and tobacco use, inadequate physical activity, political changes, less-than-optimal quality of healthcare services at primary, secondary, and tertiary levels, and the lack of preventive strategies may be the reasons (34).

This paper has limitations. Firstly, there is an issue with the data used. Although the GBD studies provide comprehensive data, it is a combination of trials and so has biases when recruiting in these trials. The accuracy of our study depends on the quality and quantity of the data input into the models. Secondly, there is an issue with the measurements. The influences of cholesterol are not merely conducted by LDL-C, so the effect of other cholesterols could not be detected.

## Conclusion

This study presented the burden caused by diseases attributed to high LDL-C, revealed the true world situation, and illuminated the focus of next-stage work. High LDL-C would continuously cause a great amount of health burden worldwide. Males suffered a greater impact of high LDL-C than females. The burden of High LDL-C was connected with age. High-income North America and Western Europe experienced a continuous decline of burden during the years. Oceania, Central Asia, North Africa and the Middle East, and Eastern Europe still had a high burden. Lower SDI regions and countries were experiencing severe challenges of health problems caused by high LDL-C when in the progress of social development and should implement more health policies.

## Data availability statement

The datasets presented in this study can be found in online repositories. The names of the repository/repositories and accession number(s) can be found below: https://vizhub.healthdata.org/gbd-results/.

## Author contributions

JZ contributed to the study design, data collection, data analysis, data interpretation, and writing of the manuscript. JW contributed to the study conduct, data collection, and writing of the manuscript. YZ contributed to the data analysis and interpretation. JX contributed to the data collection and interpretation. HG contributed to the review and data analysis. HH contributed to the review and data interpretation. PS contributed to the study concept and design, extensive data analysis, correcting of interpretation, and supervision of study implementation. TL contributed to the study concept and design, supervised the study implementation, and approved the final version of the manuscript. All authors agree to be accountable for the content of the work.

## Conflict of interest

The authors declare that the research was conducted in the absence of any commercial or financial relationships that could be construed as a potential conflict of interest.

## Publisher's note

All claims expressed in this article are solely those of the authors and do not necessarily represent those of their affiliated organizations, or those of the publisher, the editors and the reviewers. Any product that may be evaluated in this article, or claim that may be made by its manufacturer, is not guaranteed or endorsed by the publisher.

## Abbreviations

DALYs: disability-adjusted life-years
GBD: Global Burden of Disease
SEV: summary exposure value
SDI: Socio-demographic Index
LDL-C: low density lipoprotein-cholesterol
ASR: age-standardized rate
ASDR: age-standardized deaths rate
AAPC: average annual percentage change
O/E ratio: observed to expected ratio
ASCVD: arteriosclerotic cardiovascular disease
IHD: ischemic heart disease.
